# The eleventh reported case of Mulvihill-Smith syndrome in the literature

**DOI:** 10.1186/1471-2377-14-4

**Published:** 2014-01-07

**Authors:** Paulo Breinis, Flavio Geraldes Alves, Camila AE Alves, Rafael G Cintra, Débora Almeida, Priscila C Passarelli, Camila Domingues, Talita Gerbim, Régia Gasparetto, Luiz Carlos de Abreu, Vitor E Valenti, Adriana Gonçalves de Oliveira, Carlos Bandeira de Mello Monteiro, Rubens Wajnzstejn

**Affiliations:** 1 Department of Neurology, School of Medicine of ABC, Av. Príncipe de Gales, 821 09060-650 Santo Andre, SP, Brazil; 2 Departamento de de Fisiologia, Laboratório de Delineamento de Estudos e Escrita Científica, Av. Príncipe de Gales, 821, 09060-650 Santo Andre, SP, Brazil; 3 Department of Speech Language and Hearing Therapy, Faculty of Philosophy and Sciences, UNESP, Av. Hygino Muzzi Filho, 737 m, 17525-900 Marilia, SP, Brazil

**Keywords:** Case reports, Rare diseases, Neurology

## Abstract

**Background:**

The Mulvihill-Smith Syndrome was first recognized in 1975. After the recognition of the Mulvihill-Smith Syndrome, ten cases have been described.

**Case presentation:**

This article describes the eleventh case of this syndrome in a male patient, 24 years-old with short stature and microcephaly with mild cognitive impairment, deafness and allergic conjunctivitis. The patient was hospitalized several times for repeated infections, and the presence of multiple melanocytic nevi on his skin was noticed.

**Conclusions:**

Based on the entire set of signs and symptoms presented in our study, it was diagnosed the patient with Mulvihill-Smith Syndrome.

## Background

The Mulvihill-Smith Syndrome was first recognized in 1975 by David Smith Weyhe (*1926, ł1981), professor of Pediatrics at the University of Washington, and his student and future professor of Genetics at the University of Oklahoma, John Mulvihill [1].

The first case described was a male patient, 17 years-old, with short stature, microcephaly, hypodontia, recurrent infections, intellectual impairment, with multiple melanocytic nevi in the skin and insulin-dependent diabetes mellitus [1]. After the recognition of the Mulvihill-Smith Syndrome, ten cases have been described [2-10].

This article describes the eleventh case of this syndrome in a male patient, 24 years-old with short stature and microcephaly with mild cognitive impairment, deafness and allergic conjunctivitis. The patient was hospitalized several times for repeated infections, and the presence of multiple melanocytic nevi on his skin was noticed.

## Case presentation

The patient was 24 years-old male, born in Maua, Sao Paulo, Brazil. He was the eighth child of a 32 year-old woman, who had two miscarriages, did not do prenatal care and smoked during pregnancy. His parents were not consanguineous and he was delivered through vaginal delivery without perinatal complications, and born at term with adequate weight.

His neurodevelopment was normal until five years of age, when the patient, previously healthy, presented with vomiting and diarrhea lasting about a month, staying in hospital at the beginning of the clinical features to correct dehydration.

After this episode the mother told that he started to lose weight and ceased growth. He no longer understood commands and the difficulty to communicate worsened slowly. He had major mood swings, with periods of agitation, aggression and insomnia.

Since the age of six he used hearing aids. At the age of nine he was transferred to a “special school” where he remained for two years, however, he had no progress in learning.

He had several hospitalizations for diarrheal episodes and bronchopneumonia. He underwent bilateral orchiectomy at 12 years of age due to cryptorchidism.

He presented diagnosis of bilateral allergic conjunctivitis and myopia on the right eye. Dental agenesis was observed in the second premolar bilaterally and the right maxillary central incisor.

### Physical examination

**Figure 1:**
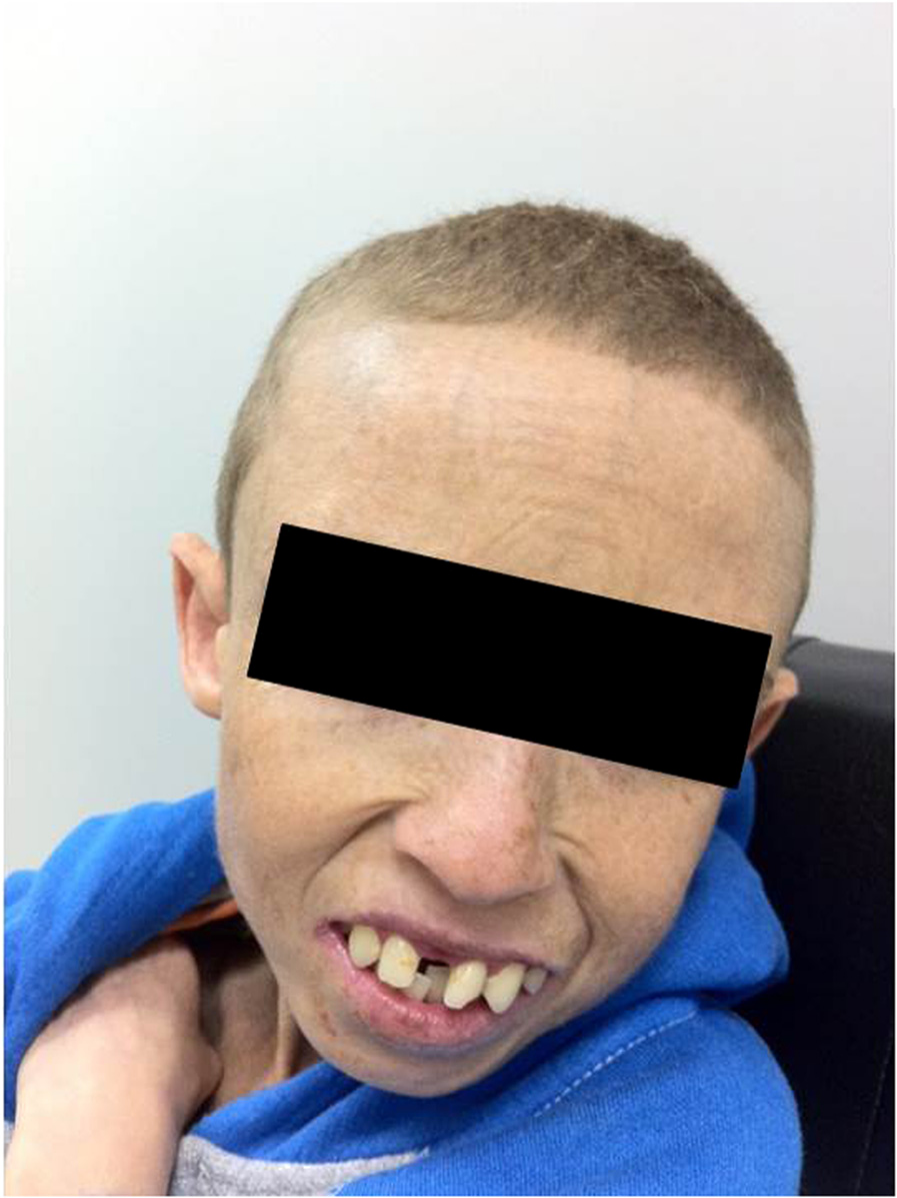
Face and trunk of the patient.

**Figure 2:**
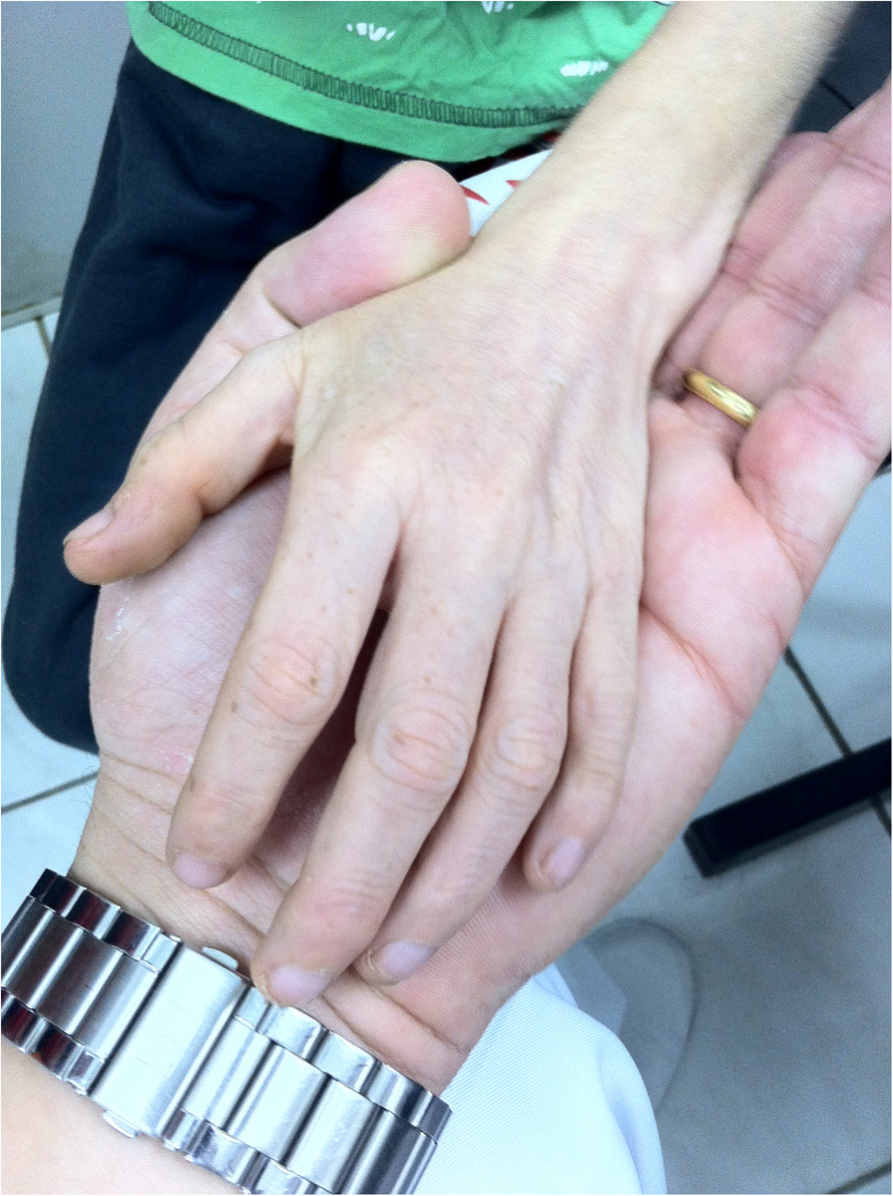
Clinodactyly on hands and feet of the patient.

Absence of subcutaneous fat was noted on his general physical examination. Skin and hair were undernourished. He had dry and aged appearance with multiple nevi on the face and trunk (Figure 1). His lips were thin and ears were prominent. He had teeth in bad condition. He had clinodactyly on hands and feet (Figure 2). There were no pulmonary, cardiac and abdominal alterations.

Our patient could keep contact with the environment through sight and hearing (a hearing aid in use). He had poor language, speech difficult to understand, in a hoarse and high voice. There was little collaboration with the examiner and extremely childish behavior. Despite no formal neuropsychological assessment was made, the patient was considered to have intellectual impairment of moderate degree.

He had hypertrophy widespread in all muscle groups. Gait, balance, deep reflexes, muscle strength, tone and cranial nerves were preserved. Coordination was difficult to assess due to no collaboration.

### Diagnostic investigation

Imaging tests such as Magnetic Ressonance Imaging (MRI) of skull and *sella turcica* and Computed Tomography (CT) were nonspecific. The X-ray revealed a bone age compatible with the age of ten.

The metabolic tests performed were total cholesterol and fractions, triglycerides, TSH, T4, serum GH, GOT, GPT, Gamma GT, amylase, blood glucose, blood gas analysis, screening of inborn errors of metabolism blood and urine. Were also analyzed blood count, electrolytes, renal function, ANA, RF, hemoglobin electrophoresis, serology for CMV, Rubella, Toxoplasmosis, Hepatitis B and Epstein Barr, CD3, CD4, CD8, IGF-1 and IGFBP.

His blood count revealed lymphocytosis, the dosage of growth hormone and IGF-1 were below the reference value. The CD4 counts were at the lower limit and presented a very high parameter of immunoglobulin G to infection with Epstein Barr VCA. All other metabolic tests results were shown normal.

A standard resolution karyotype was also normal, and no further genetic investigations were performed. The hypothesis was diagnosed based on clinical criteria.

## Discussion

The Mulvihill-Smith syndrome is a rare and complex genetic disorder, which involves different systems and organs. The description of a patient born to consanguineous parents [9], and the presence of the syndrome in both males and females, suggests of autosomal recessive inheritance. However, it is worth noting that our patient the only one affected offspring of eight offspring. This would still compatible with recessive inheritance, possibly with one mutation inherited from either one parent and the other mutation arisen de novo. The causative gene has not been identified so far [2].

Since the description of the first case of Mulvihill-Smith Syndrome, a number of signs and symptoms have been reported (Table 1).

**Table 1:** Cases described

	**Mulvihill and Smith (1975)**	**Shepherd (1971) and Elliot (1975)**	**Wong et al. (1979)**	**Baraitser et al. (1988)**	**Ohashi et al. (1993)**	**Bartsch et al. (1994)**	**De Silva et al. (1997)**	**Ferri et al. (2005)**	**Yagihashi et al. (2009)**	**Fuhler-Stiller et al. (2010)**	**Gebin et al. (2011)**
**Sex**	M	M	F	M	F	M	M	F	F	M	M
**Age**	17	3,4	14	7	30	20	4	25	28	16	24
**Birth weigth**	1800 g	1890 g	1800 g	1880 g	2700 g	3340 g	2600 g	NR	2570 g	2290 g	2930 g
**Consanguinity**	-	-	-	-	+	-	-	-	-	-	-
**Short stature**	+	+	+	+	+	+	-	+	+	+	+
**Microcephaly**	+	+	+	+	+	+	+	+	+	+	+
**Pigmented nevi**	+	+	+	+	+	+	+	+	+	+	+
**Loud and raucous**	+	NR	NR	+	-	+	+	+	+	+	+
**>Facial fat**	+	NR	+	+	+	+	+	+	+	+	+
**Hypertelorism**	NR	+	+	-	+	+	-	-	-	+	+
**Alopecia**	+	+	NR	NR	-	-	-	-	+	-	-
**Brachydactyly**	+	+	NR	NR	+	+	-	NR	+	NR	-
**Visual change**	-	-	-	NR	-	+	-	+	+	+	+
**Hypospadias**	+	+	+	+	+	+	+	NR	NR	NR	+
**Diabetes**	+	-	-	-	-	-	-	-	+	+	-
**Recurrent infections**	+	+	+	-	+	+	+	+	+	+	+
**Deafness**	+	+	+	+	+	+	-	+	+	+	+
**Development of tumor**	-	-	-	-	-	Gastric	-	Tongue	Pancreas	Melanoma	-
**Mental retardation**	+	+	-	+	+	+	-	+	+	+	+
**Sleep disorder**	NR	NR	NR	NR	NR	NR	NR	+	+	NR	+

All cases describe microcephaly and pigmented nevi on skin. Short stature is absent only in one case [4,5]. Four previously reported patients also exhibited the early onsets of tumors: signer ring cell carcinoma of the stomach in a 23 year-old patient [4,5], and squamous cell carcinoma of the tongue in a 25 year-old patient [5]. Pancreatic cancer was described in a female patient of 28 years of age [2], and skin melanoma have also been described.

Sleep disorders are described in three cases [2,7,8]. The loud and raucous (high-pitched voice) observed in this case and in seven cases, perhaps reflects the abnormal structures of the face [6].

Ohashi et al. [9] described urogenital abnormalities in other cases, with cryptorchidism, hypospadias, descended under test, and amenorhoea. The cryptorquidia is present in this case [9].

In almost all cases low levels of immunoglobulins and T and B cells were found. This information associated with the presence of rhinitis and allergic conjunctivitis and recurrent infections, strengthens the idea that immunodeficiency can be one of the characteristics of the syndrome [4,5].

Mulvihill-Smith Syndrome has been associated with several levels of intellectual impairment, so far present in every case described. Our patient also has clinodactilia also reported in four other cases. Only one case was not identified with deafness [6].

## Conclusion

Given the identified cases and the entire set of signs and symptoms described in our case we clinically diagnosed the patient with Mulvihill Smith Syndrome.

## Consent

Written informed consent was obtained from the patient for publication of this case report and any accompanying images. A copy of the written consent is available for review by the Editor-in-Chief of this journal.

## Competing interests

The authors declare that they have no competing interest.

## Authors’ contributions

All authors participated in results collection, study design and manuscript draft. All authors agreed with the final version of the manuscript.

## Pre-publication history

The pre-publication history for this paper can be accessed here:

http://www.biomedcentral.com/1471-2377/14/4/prepub
